# Supplementary material for: Plant Photocatalysts: Photoinduced Oxidation and Reduction Abilities of Plant Leaf Ashes under Solar Light
Source: Nanomaterials (Basel). 2023 Aug 6;13(15):2260. doi: 10.3390/nano13152260 (PMC10421452; doi:10.3390/nano13152260)
Supplement: Supplementary file 1 [file nanomaterials-13-02260-s001.zip › nanomaterials-2488287-supplementary.pdf]

Supporting Information

Plant Photocatalysts: Photoinduced Oxidation and Reduction Abilities of Plant Leaf Ashes under Solar Light

Xiaoqian Ma [1,†], Jiao He [1,†], Yu Liu [2], Xiaoli Bai [1], Junyang Leng [1], Yi Zhao [1], Daomei Chen [2,*] and Jiaqiang Wang [1,2,*]

[1] School of Chemical Sciences & Technology, Yunnan University, Kunming 650091, China; 18290855825@163.com (X.M.); hejiao@ynu.edu.cn (J.H.); m18213412529@163.com (X.B.);
ljy262@163.com (J.L.); m18788591505@163.com (Y.Z.)

[2] School of Materials and Energy, Yunnan University, Kunming 650091, China; liuyu19990204@163.com

* Correspondence: dmchen@ynu.edu.cn (D.C.); jqwang@ynu.edu.cn (J.W.); Fax: +86-871-65031567

† These authors contributed equally to this work.

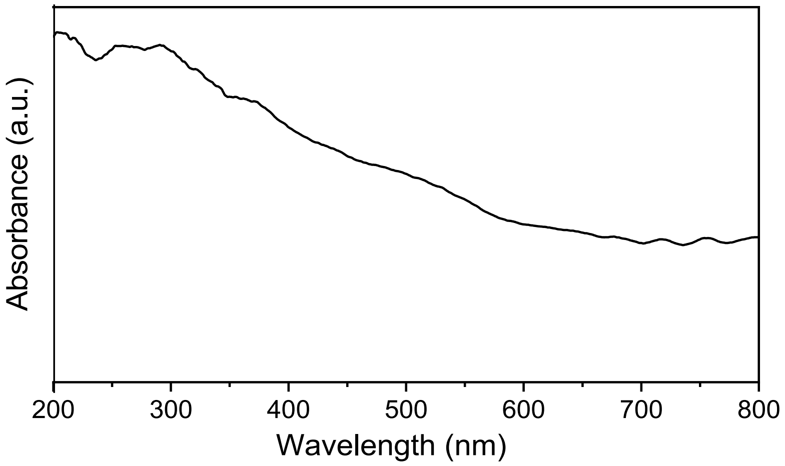

Figure S1. UV−vis diffuse reflectance spectra of couchgrass ash.

Table S1 Comparison of the main elements content of the garlic sprouts (stem) determined by XRF and XPS.

Name	The main element content (mass %)							
	K	Cl	O	Ca	C	Mg	Si	B
XRF-garlic sprouts (stem)	27.64	22.70	28.06	6.77	6.24	1.85	0.23	2.11
XPS-garlic sprouts (stem)	2.34	0.61	54.05	10.93	22.86	5.78	4.36	2.27

Table S2 Summary of textural properties of three plant ashs.

Samples	S_{BET}(m^2g^{-1})	Pore volume(cm^3g^{-1})	Pore size(nm)
garlic sprouts (leaf)	5.82	0.0097	41.52
Chinese leek (leaf)	3.62	0.0048	36.08
garlic sprouts (stem)	3.69	0.0054	34.61
Chinese leek (stem)	1.51	0.0024	20.69

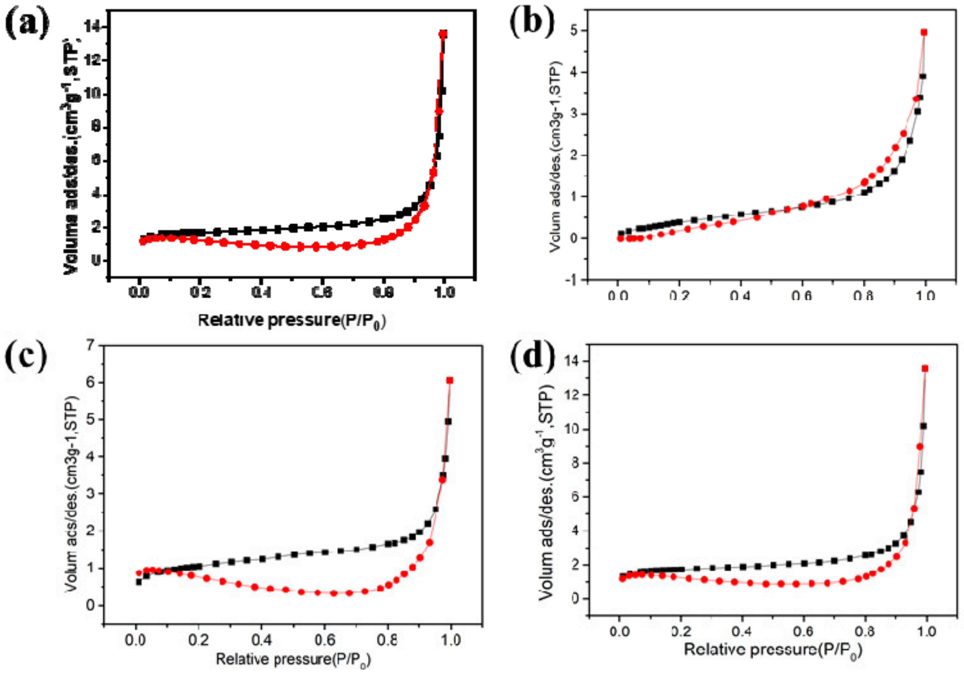

Figure S2 N_2 adsorption/desorption isotherm distribution of garlic sprouts (leaf) ash(a), Chinese leek (leaf)(b), garlic sprouts (root) ash(c) and Chinese leek (stem)(d).
